# Detoxification Assays of Tunisian Tannery Wastewater under Nonsterile Conditions Using the Filamentous Fungus* Aspergillus niger*

**DOI:** 10.1155/2019/9020178

**Published:** 2019-06-03

**Authors:** Raouia Boujelben, Mariem Ellouze, Sami Sayadi

**Affiliations:** ^1^Laboratory of Environmental Bioprocesses, Centre of Biotechnology of Sfax, University of Sfax, PO Box 1177, 3018 Sfax, Tunisia; ^2^Center for Sustainable Development, College of Arts and Sciences, Qatar University, Doha 2713, Qatar

## Abstract

The ability of* Aspergillus niger *strain to reduce organic and mineral pollution as well as the toxicity of two tannery wastewaters, the unhairing effluent (UE) and the final effluent (FE), taken from a local Tunisian tannery and under nonsterile conditions, was studied. Raw effluents show alkaline pH ≥11; thus experiments were carried out at initial pH values and at pH adjusted to 6. Characterization of effluents also revealed high salt levels (EC > 17 mS/cm) and high organic matter content (25 g/L for the UE and 7.2 g/L for the FE) but a low biodegradability since BOD_5_ did not exceed 2.5 and 1.25 g/L for the UE and the FE, respectively. The results of the biological treatment showed that* A. niger* was able not only to grow at high pH and salinity values, but also to reduce organic and mineral pollutant load. After treatment, the COD reduction for the UE reached 90% and 70% at pH=6 and at initial pH (12.13), respectively. For the FE, the decrease of COD values reached 75% at pH=6 and 64% at initial pH (11.64). Monitoring of mineral pollution levels showed a reduction in chromium (Cr) concentrations reaching 70% for the FE. This was reflected by an increase of the biomass of* A. niger* from 9.25 g/L (control) to 9.84 g/L for the FE. To confirm the efficiency of the biological treatment using* A. niger, *phytotoxicity (tomato seeds) and microtoxicity (*Escherichia coli* and* Bacillus subtilis)* tests were carried out. Results of this monitoring showed an important decrease in the toxicity levels for both effluents.

## 1. Introduction

Environmental pollution is turning into a worldwide problem in which water pollution is a critical issue since water is used for different purposes [1]. Thus, the management of water pollution is considered as one of the major challenges for environmentalists [2]. This pollution is generally due to the quick rise of industrial activities which extremely increased the release of toxic wastes into water bodies along with ground water [3]. Causing disturbance to the natural bodies and their ecosystems, water pollution leads to climatic changes, water level reduction, and other negative impacts [2].

Among all industries, the tanning sector is considered as one of the main consumers of water and consequently the most pollution generator [4]. In fact, the tanning process aims to transform putrescible material “animal skins” into stable and imputrescible material “leather” [5]. This transformation consists essentially of eliminating the interfibrillar matter of the raw skin through a series of pretanning operations, also known as beamhouse operation, rendering the skins rot-resistant by tanning, and adding to them aesthetic qualities during posttanning and finishing steps [6]. During the leather making process, 25 up to 80 m^3^ of wastewater are generated per ton of raw material [7]. These wastewaters are highly turbid, colored, and foul-smelling [8]. Besides, they contain high levels of natural and synthetic organic dyes, organic nitrogen, sulfates, sulfides, and heavy metals, mainly chromium [9, 10]. The composition of organic pollutants in tannery effluents is complex. Proteins, mainly collagen and their hydrolysis products, especially amino acids derived from the skin, are predominant, while others, such as fats, are at low concentrations [11]. Tanneries also use compounds like aliphatic amines, nonionic surfactants, oils, and pigments. The main odorous gases in tannery effluents are volatile organic compounds (VOCs), hydrogen sulfide (H_2_S), and ammonia (NH_3_) [12]. High concentrations of such organic and inorganic pollutants with low biodegradability represent actually a serious technological and environmental challenge [13]. On the other hand, the toxicity of tannery effluents was reported in several studies [14–16]. Most of these studies revealed that the effects of tannery wastewater are devastating not only to humans [17] but also to birds [18], fish [19], plants [20], amphibians [21], and algae and daphnia [22, 23]. Furthermore, toxicity of tannery effluents to mice was investigated [16, 24, 25]. Rabelo* et al.* [25] pointed that chronic exposure of Swiss mice to tannery effluents causes histological changes in their livers. However, Mendes* et al.* [16] proved that even a short-term exposure of male and female C57Bl/6 J mice to tannery effluents led to a deficit of their olfactory response, to a low response to predators as well as a partial memory deficit in the task of inhibitory avoidance.

Besides, due to the unawareness of the toxic effluents they are dealing with, tannery workers are exposed to the risk of developing diseases related to genetic damage [17].

All of these researches confirm the high toxicity of tannery effluents, basically due to their chemical complexity [24]. Therefore, tanneries are constrained to treat their effluents in order to minimize the environmental impacts. Physical and chemical methods including coagulation, flocculation, oxidation, and adsorption are commonly applied for tannery wastewater treatment [13]. It was reported that coagulation/flocculation reduces COD to only 37% and suspended solid by almost 40% but eliminates chromium up to 99% [26]. Other studies focused on membrane treatment showed the efficiency of this process in the removal of turbidity (98%) using microfiltration and COD (90%) with ultrafiltration [27]. The nanofiltration eliminates more than 90% of COD and sulfates according to Ahmed* et al.* [28]. However, physicochemical treatments are efficient but not cost effective in terms of energy and reagent utilization. Moreover, they are not ecofriendly methods and they generate large quantities of sludge which renders waste disposal problematic [29].

As a result, special attention is given to biological processes since they are generally cost effective and environmentally friendly [30]. There are some studies which have been conducted employing bacteria for remediation of the components of tannery wastewater [31] or fungal mycelia as bioabsorbent [32, 33]. The ability of fungi to corporate, to adapt, and to grow under extreme conditions such as pH, high salinity, nutrient availability, and the presence of heavy metals or other inhibitory compounds, is what makes their uses to treat complex effluents more frequently attractive [34, 35].

The aim of this work is to evaluate the treatment efficiency of two tannery effluents (the UE and the FE) generated from the unhairing and the final steps of the tanning process using the filamentous fungus* A. niger* at different pH values and under nonsterile conditions. The work also attempts to study the detoxification capability of the strain by monitoring the toxicity of the effluents before and after fungal treatment using seed germination test (*Lycopersicon esculentum*) and microtoxicity test (*Escherichia coli* and* Bacillus subtilis)*.

## 2. Materials and Methods

### 2.1. Samples

Effluent samples were obtained from a local Tunisian tannery in Sfax city. Two effluents were collected from two different steps of the leather making process: the UE was collected from the unhairing bath where skins are immersed in lime to eliminate the epidermis and the hairs; the FE was collected from the final discharge bath where effluents from beamhouse, tanning, and retanning operations are collected together. No previous pretreatment was carried out for both effluents. All samples were collected at three different periods. For each measure, values are the mean of triplicate. The values given in the tables are the means of the three values corresponding to the different periods.

Samples were stored at 4°C for no longer than 4-5 days; otherwise they are frozen at -20°C. In most of the cases, fresh samples were immediately analyzed.

### 2.2. Physical and Chemical Characterizations

The pH and the electrical conductivity (EC) were determined using a pH meter model Istek-NeoMet and a conductivity meter model CONSORT C831, respectively.

Chemical oxygen demand (COD) was estimated as described by Knechtel [36]. A total reflux digestion was achieved by reaction with H_2_SO_4_ and potassium dichromate at 150°C for 2 h. Given the presence of salts in tannery effluents, COD is determined by dosing with a solution of Mohr salt (N/40) with the presence of ferroin as indicator. Biological oxygen demand (BOD_5_) was determined after 5 days by the manometric method with a respirometer (BSB Controller Model 620 T (WTW)) [37]. Total solids (TS) and total suspended solids (TSS) were determined by weighing samples before and after drying overnight at 105°C. Volatile solids (VS) and volatile suspended solids (VSS) were analyzed by loss on ignition at 600°C for 2 h, according to APHA [37]. The total nitrogen content (TKN) and N–NH_4_^+^ were analyzed as described in Kjeldahl-N method [38]. Heavy metals concentrations were determined by flame atomic absorption spectrometry of samples digested with an acid mixture of HCl and HNO_3_.

The soluble protein content was determined using the Bradford method [39]. The determination of fats and sulfide (H_2_S) content in the samples was performed according to the method of Cord-Ruwisch [40], using hexane as a solvent.

### 2.3. Strain and Culture Conditions

*A. niger *strain (CTM 10099) used in this study was taken from the *«*Tunisian Collection of Microorganisms (TCM)*»* of the Centre of Biotechnology of Sfax (CBS Sfax, Tunisia). The strain was maintained on potato dextrose agar slants (PDA) at 30°C with a periodic transfer.

For experiments, an acclimated strain of* A. niger *was used. The acclimated fungus was prepared by growing on PDA dishes containing effluents (15%). Spores from acclimated strain of* A. niger *were produced by growth in PDA for 5 days at 30°C and harvested by suspending with a sterilized solution of 0.1% of Tween 80.

Aerobic biological treatment of tannery wastewater was carried out under nonsterile conditions. In the first set of experiment, pH of both effluents was not modified. For each effluent, basal medium (pH= 5.5) containing per liter 0.5 g (NH_4_)_2_SO_4_, 2 g KH_2_PO_4_ in the presence of 0.1% of a sterilized glucose solution, was added. In the second set of experiment, the same concept described above was done with exception that the pH was adjusted to 6.

Afterward, 0.2 mL of the spore solution was transferred to the production culture containing 15% of raw wastewater. The inoculated flasks were continuously shaken on a rotary shaking incubator operating at 160 rpm and 30°C for 7 days.

Since effluents were used under nonsterile conditions, positive control with only effluent was used under the same conditions mentioned above.

### 2.4. Phytotoxicity Assay

The phytotoxicity of the tannery effluents was measured by the determination of the germination index (GI) [41] using tomato seeds (*Lycopersicon esculentum)*. Ten seeds were placed in standard Petri dishes covered with filter paper. Seeds were irrigated with 5 mL of the respective samples and the dishes were incubated in the dark for 5-7 days at 25°C. Control Petri dishes were irrigated with tap water.

For all samples, seedling phytotoxicity was measured using different dilutions of raw and treated effluent samples (10, 20, 50, and 100%). Dilutions were chosen based on some literature studies, especially those of Mlaik* et al.* [20], and also on our preliminary assays in which we found that 30% was nearly the same as 20% and 40% gives very similar results to those of 50%. For proportions from 60% to 90%, there was a high inhibition level of the germination seeds; that's why these proportions were not mentioned. But the use of 100% (raw effluents) is to confirm the total inhibition of germination seeds.

Each analysis was run in three replicates and mean values of germination index are quoted as results. The GI was defined as follows:

(1) $$\begin{array}{c} GI\left(\;\%\;\right)=\left(\;\frac{\text{NGS sample}\times \text{ALR sample}}{NGS\;\;blank\times ALR\;\;blank}\;\right)\times 100 \end{array}$$

where NGS is the number of germinated seeds and ALR is the average root length of seeds.

### 2.5. Microtoxicity Assay

Microtoxicity tests were performed in order to assess the toxicity levels of both untreated and treated effluent. The microtoxicity was studied using* E. coli *and* B. subtilis *strains, reported as the most sensitive microorganisms [42], cultivated on a nutrient broth (NB) as controls and incubated at 37°C. Effluents were used at final concentrations of 20% and 100% in the NB. The bacterial growth was assessed by optical density (OD) measurement at 600 nm recorded at 2 h intervals for 10 h. Since effluents were used under nonsterile conditions, positive control with only effluent (before and after treatment) was used under the same conditions mentioned above.

### 2.6. Statistical Analysis

Values given were represented as standard deviation (means ± SD) of three independent replicates. All data were statistically analyzed by two-way ANOVA using Graphpad Prism software (version 6). Tukey's multiple comparisons test with a significance level of *α* < 0.05 was applied.

## 3. Results and Discussion

### 3.1. Tannery Wastewater Characterization

Two effluents from a Tunisian tannery were used in this study: the unhairing effluent (UE) and the final effluent (FE). The average values of different physical and chemical parameters of the collected samples are presented in Table 1.

The unhairing and the final wastewater present high pH values reaching 12.13 and 11.64, respectively. Such alkaline pH in both effluents is due to the liming step in which hair and flesh are removed from the skin through the use of lime and sodium sulphide [43]. Comparing with the Tunisian standards of discharges “NT 106.002” [44], both effluents have nonadmissible pH values. Analysis indicated a high EC, exceeding 17 mS/cm, showing a high salt level reaching 14.8 and 15.75 g/L for the UE and the FE, respectively. These high EC values indicate that salts are present at high levels in both effluents [43]. Moreover, both samples contain high COD values: 25 g/L for the UE and 7.2 g/L for the FE. The low BOD_5_/COD ratio, which was equal to 0.1 for the UE and 0.17 for the FE, confirms the recalcitrance of the effluents and their low biodegradability. According to the study made by UNEP [45], BOD_5_ and COD values in the tannery effluent are generally higher than the tolerance limits for inland surface water.

Additionally, UE was characterized by high contents of total Kjeldahl nitrogen reaching 2.63 g/L, compared to the FE (0.448 g/L). These values exceeded the values suggested by Tunisian standards for discharges “NT 106.002” [44]. The high nitrogen content in the UE is possibly due to the fact that several components in tannery effluent contain nitrogen as part of their chemical structure. The most common are ammonia (resulting from the use of ammonium sulphate during the deliming step) and the nitrogen contained in proteinaceous materials (from liming/unhairing operations) [46]. It was reported that the amount and the composition of total nitrogen in tannery effluents vary according to the type of leather treated, the amount of chemicals used during treatment, water consumption, and other factors such as the duration of the process [43].

On the other hand, experiments revealed the presence of the toxic gas H_2_S in the UE. This is due to the use of sodium sulphide in the liming step. Once it's mixed with acid wastewater from the pickle liquor step and through the activity of sulfate reducing bacteria present in tannery effluents, this gas is generated [47]. Since H_2_S is known by its toxicity and odor even at low concentration [48, 49], an efficient removal is required.

Due to the use of chromium in the tanning process, analyses revealed high value in the FE (7.02 mg/L) exceeding the Tunisian standards of discharges (≤ 0.1 ppm) for this component. Akan* et al.* [50] reported related results for heavy metals concentrations in tannery effluents. They stated that the mean concentrations of metals in tannery effluents were high compared to standard limits.

### 3.2. Biological Detoxification of Tannery Wastewater Using* A. niger*

Treatment assays of tannery wastewater with pure cultures of* A. niger *were carried out using 15% of raw nonsterile effluent. Even though the UE and the FE present alkaline pH values (above 11) and contain a high concentration of salts (salinity nearby 15 g/L),* A. niger *was able to grow and to tolerate such extreme conditions. It was reported that this strain was able to tolerate such extreme conditions when treating olive mill wastewaters and landfill leachates [34, 51]. Indeed, the ability of other fungal strains, including* Trametes trogii* and* Phanerochaete chrysosporium* to tolerate high pH and salinity values, was proved in previous studies [52, 53].

The challenge in this case study was to successfully treat tannery wastewater with fungi under nonsterile conditions because it's known that tannery wastewater contains high microbial community that might lead to faster and higher contamination, beside the high pollution load that could inhibit fungal growth. Knowing that so far, most of the treatments of real wastewater under nonsterile conditions have been carried out for textile wastewater [54] or with the addition of spiked contaminants [55].

#### 3.2.1. Organic Pollutants Removal (COD and BOD_5_)

**Figure 1:**
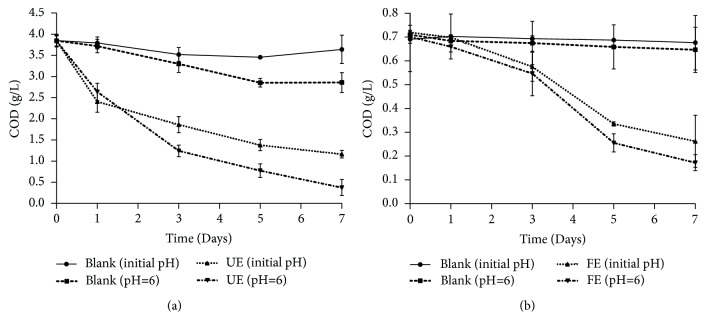
COD removal during treatment assays with* A. niger *of the UE (a) and the FE (b). Results are expressed as the mean of three independent tests ± standard deviation.

During the fungal treatment assays of the UE, COD removal was more important within the first two days of treatment; more than 50% of initial COD was eliminated (Figure 1(a)). After 7 days of incubation, reduction of COD reached 70% and 90% for pH=12 and pH 6, respectively. For the FE, treatment assays showed that the abatement values of COD reached 64% and 75% for pH= 11.64 and 6, respectively (Figure 1(b)). The decrease in the COD points to the reduction of biologically oxidable and inert organic materials as a result of their degradation by the fungus [56]. This shows that, even under such stressful conditions and the nonsterile conditions (existence of native microorganisms),* A. niger* was able to develop various mechanisms in order to cope with the adverse conditions [57]. Nearby, COD removal was clearly influenced by the pH and the composition of the effluent used. As a consequence, the removal was more significant at pH=6, especially for the treatment of the UE (*p* value < 0.0001).

Apart of monitoring COD removal, BOD_5_ values were also determined after the treatment assays of each effluent. As shown in Table 2, reduction of BOD_5_ reached 90% for the UE but didn't exceed 60% for the FE, when pH was 6. This reveals that biodegradability of both wastewaters was enhanced after fungal treatment while reduction was more important during the treatment of the UE.

Besides the effect of pH, results show the influence of chemical composition of the effluent used (UE or FE) on the treatment efficiency. Although no characterization of organic pollutants was carried out in this study, previous studies reported the profile of organic and inorganic compounds in tannery effluents. According to Mlaik* et al.* [20], UE contains high organic load, lime, and sodium sulfide since they are used during liming-unhairing operation. Also, Mlaik* et al.* [58] pointed to the presence of many hydrocarbons, including saturated (C16:0, C18:0) and unsaturated fatty acid methyl esters as well as long- and short-chain alkenes in the UE. For the FE, many studies revealed the presence of xenobiotics [59], phenolic and long chain aliphatic compounds [60], other organic compounds such as 1-nonadecene and 2-phenylethanol [61], phthalates [24], chlorinated phenols, chromium, and formaldehyde resins [17]. However, the chemical composition of tannery effluents depends on the size of the tanning industry, the chemicals used, the raw material, and the technology adopted during the leather making process [62]. But, according to Tadesse and Guya [17], chlorinated phenols (3,5-dichlorophenol) and chromium were usually detected in all tannery effluents, which maybe explains the inhibitory effect of the FE during the fungal treatment, compared to the UE.

#### 3.2.2. Inorganic Pollutants Removal

Along with monitoring the removal of organic pollutants and due to the high levels of some heavy metals and other minerals in raw tannery wastewater, the ability of* A. niger* to reduce these pollutants was studied. Results are presented in Table 2. Analysis showed that concentrations of mineral pollutants decreased at the end of the fungal treatment: chromium removal exceeded 64% in the case of the FE at pH=6. These results suggest that the reduction of mineral pollution is mainly due to the ability of* A. niger* to accumulate metals in its mycelia, a statement reported in several works [32, 63]. This was deduced by the increase in the biomass of* A. niger *from 9.25 g/L (control) to 9.84 g/L for the FE. However, despite the significant decrease of Cr levels, it remained above the values imposed by the NT 106.02 and fixed at 0.1 ppm.

### 3.3. Toxicity Monitoring

During this study, biological treatment was run under nonsterile conditions, as indicated previously. The main drawback usually reported in such a case is the overtaking of the inoculated fungus by native microorganism present in the wastewater. Thus, some toxicity assays were tested to confirm the ability of* A. niger *to reduce the effluent's toxicity by removing the organic and inorganic pollutants in both effluents.

#### 3.3.1. Phytotoxicity Assay

**Figure 2:**
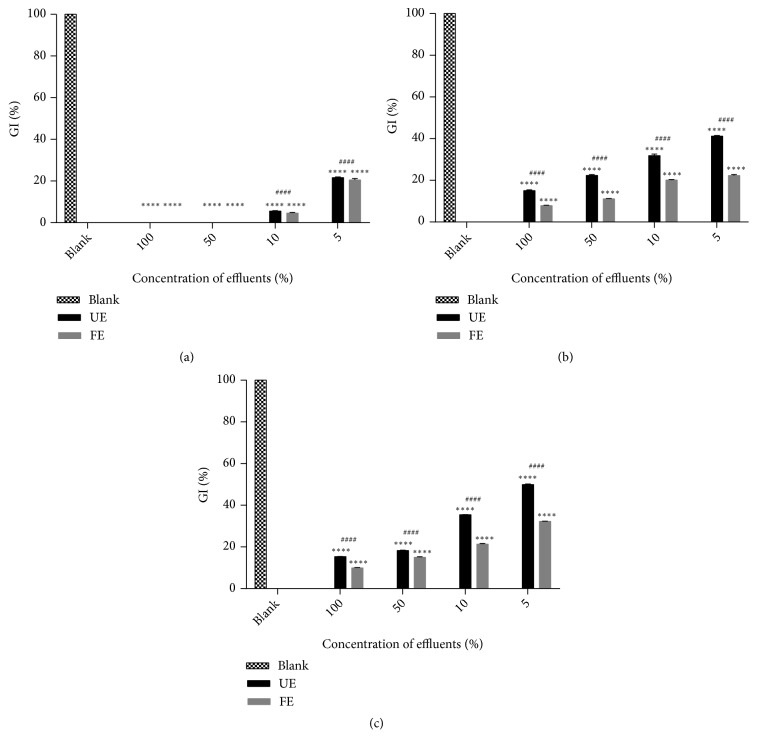
Phytotoxicity test: germination index (%) of* L. esculentum *grown on raw effluents (a) and on treated effluents at initial pH (b) and at pH=6 (c). Values given represent the mean of three replicates. The asterisk indicates* p* value Blank versus* p* value UE or FE (*∗∗∗∗*p < 0.0001). The sharp indicates* p* value UE versus* p* value FE (####p < 0.0001).

Phytotoxicity test was carried out using tomato seeds of* L. esculentum*. Different concentrations of effluents were prepared and tested (100, 50, 10, and 5%). The results of this monitoring, illustrated in Figure 2(a), show that both effluents were very toxic since there was a total inhibition of seed germination for raw and diluted effluents (up to 50%). Even at 5%, both wastewaters remain toxic since the GI didn't exceed 22%. The very high toxicity of raw effluents is probably due to high levels of organic and inorganic pollutants, but also due to the high salt level. Vicente* et al.* [64] proved that high EC is known to have inhibitory effects on seed germination. In fact, high salt concentrations in raw effluent lead to high osmotic pressure, which could cause the suppression of seed germination and a delay in the emergence of seedlings [65]. Thus, results presented in Figures 2(b) and 2(c) revealed that, after treatment assays, GI were slightly higher in the case of UE perhaps because it presents a lower EC than the FE. Moreover, the treated effluent had a valuable and stimulatory effect on seed germination. These results highlighted a relative tolerance in the case of the UE. This tolerance was slightly more significant when pH was adjusted to 6, since the GI reached 50 and 32% for the UE and the FE, respectively (*p* value < 0.0001), whereas it was about 41 and 22% for effluents at initial pH values > 11. Such growth promotion could be attributed to the fact that the toxic substances were diluted to a low level [66]. Moreover, the phytotoxicity test showed that* L. esculentum *was more sensitive to the FE since it has higher levels of EC and heavy metals, especially the chromium. In fact, Sahu* et al*. [67] revealed that when more than 2 ppm of Cr is present, water becomes unsuitable for the growth of crops. In this case of study, the Cr concentration was around 7.02 ppm in the FE which further explains the inhibition of the seed germination in the raw effluent. Also, the study done by Rao* et al.* [68] showed that not only the presence of Cr but also high level of BOD, COD, and other nutrient contents in the effluent have an inhibitory effect on seed germination and on seedling growth.

#### 3.3.2. Microtoxicity Assay

**Figure 3:**
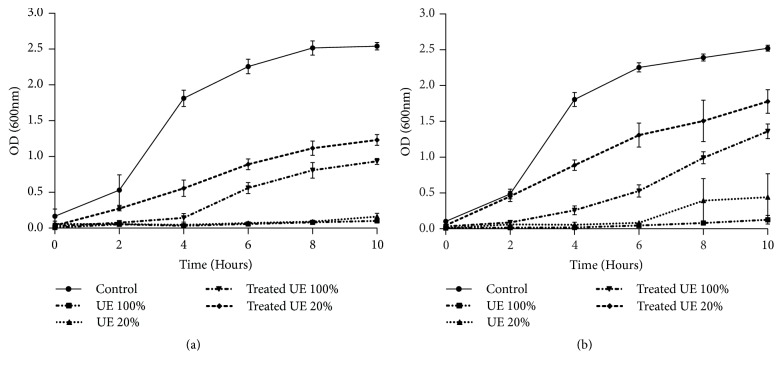
Microtoxicity monitoring: growth of* E. coli *on the UE before and after fungal treatment at initial pH (a) and at pH=6 (b). Results are expressed as the mean of three independent tests ± standard deviation.

**Figure 4:**
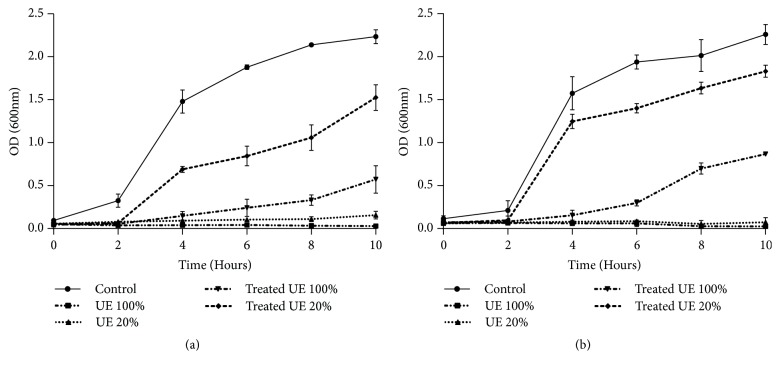
Microtoxicity monitoring: growth of* B. subtilis *on the UE before and after fungal treatment at initial pH (a) and at pH=6 (b). Results are expressed as the mean of three independent tests ± standard deviation.

**Figure 5:**
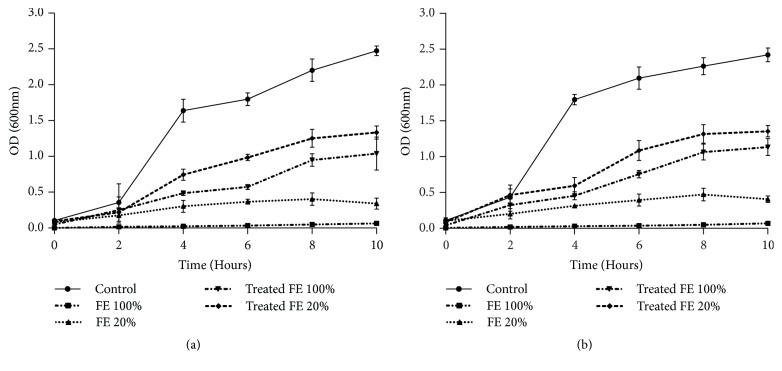
Microtoxicity monitoring: growth of* E. coli *on the FE before and after fungal treatment at initial pH (a) and at pH=6 (b). Results are expressed as the mean of three independent tests ± standard deviation.

**Figure 6:**
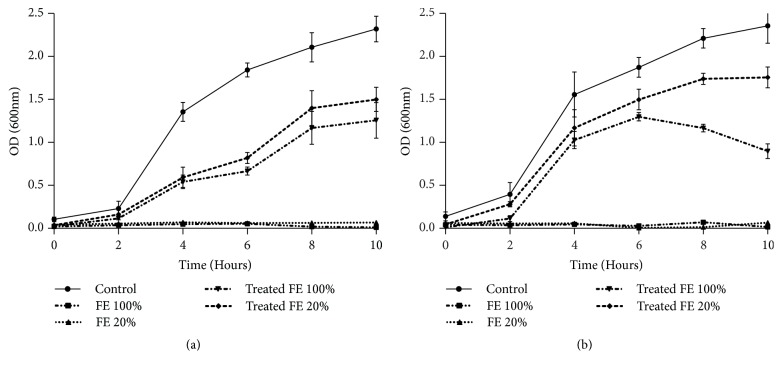
Microtoxicity monitoring: growth of* B. subtilis *on the FE before and after fungal treatment at initial pH (a) and at pH=6 (b). Results are expressed as the mean of three independent tests ± standard deviation.

The relative sensitivity of the bacterial strains* E. coli *and* B. subtilis *towards treated and untreated tannery effluents was studied. Microtoxicity assay was done on the nutrient broth medium as control and on nutrient broth medium supplemented with raw, diluted, and biologically treated effluents. The results of this monitoring are presented in Figures 3, 4, 5, and 6. The microtoxicity study showed that the untreated effluents totally inhibited the growth of* E. coli* and* B. subtilis*. As was expected, raw wastewaters were very toxic. However, the growth curves of the two bacterial strains on the media supplemented with biologically treated effluents exhibited a decrease in the toxicity levels. Moreover, both treated effluents were less toxic since the bacterial growth was not inhibited. These results suggested that fungal treatment using* A. niger *led to an important decrease of the toxicity and could be consequently efficient for tannery wastewater treatment even under nonsterile conditions. In fact, as mentioned in Figures 3, 4, 5, and 6, kinetics of growth indicated different slopes; the growth of bacterial strains decelerates when high levels of raw effluents were added to the medium. Even with a five-time diluted effluent, a considerable inhibition of both* E. coli* and* B. subtilis *growth was obtained. However, after fungal treatment assays, growth patterns of the bacterial strains were sort of similar to the growth patterns on NB medium, especially with 20% of treated effluents. The best results of this monitoring were obtained for* B. subtilis *strain where pH was adjusted at 6, as shown in Figures 4(b) and 6(b). Therefore, it can be stated that the variation of pH values and the chemical composition of the effluents strongly influenced the behavior of both bacterial strains. As shown in Figures 3 and 4, the behavior of* E. coli* and* B. subtilis *was different when treating the UE with pH variation: for both strains, the growth was better for treated effluents when pH was 6. Besides, these results demonstrate that* E. coli *seems to be more sensitive than* B. subtilis*. Indeed, the difference in sensitivity was more evident for the FE at pH=6 where* B. subtilis *was slightly inhibited (25%) as mentioned in Figure 6(b), while the inhibition was more noticeable for* E. coli*, exceeding 44%, under the same conditions (Figure 5(b)).

Overall, the ability of* A. niger* to reduce organic and mineral pollution as well as the toxicity of tannery effluents under nonsterile conditions was confirmed and could be a good alternative for scaling up this treatment after several optimizations for industrial bioreactor scale. According to the literature, very few studies reported the fungal treatment of tannery effluent under nonsterile conditions. Besides, the tannery effluent treatment plants commonly use the coagulation/flocculation, which is relatively a much expensive method that produces sludge with high level of chemicals. Thus, this biological process could be an ecofriendly and an economic alternative to solve the excessive use of chemicals since chromium could be regenerated and reused.

## 4. Conclusion

In the present study, raw tannery effluents showed alkaline pH ≥11, high salt levels (EC > 17 mS/cm), and high organic matter content with 25 g/L for the UE and 7.2 g/L for the FE. For the FE, analyses also revealed a high value of chromium reaching 7.02 mg/L. According to phytotoxicity and microtoxicity tests, raw and twice diluted effluents were toxic since they completely inhibited the GI of* L. esculentum *seeds and the growth of* E. coli* and* B. subtilis *strains. The biological treatment using* A. niger *was efficient since it led to a significant decrease of organic and inorganic pollutants. Genuinely, COD removal efficiency was more significant at pH=6, reaching 90% for the UE and 75% for the FE, on the 7th day (*p* value < 0.0001). The efficiency of the treatment closely depended on pH values and chemical effluent composition. Monitoring of mineral pollution levels showed an important reduction in chromium concentrations reaching 70% for the FE at pH=6. According to phytotoxicity tests for the treated samples, the improvement in the germination index was more important for the UE at pH=6 with GI reaching 50% while it was about 32% for the FE. For the microtoxicity tests assays, the growth curves of* E. coli* and* B. subtilis *on the treated samples exhibited a decrease in the toxicity levels. However, the difference in sensitivity was more evident for the FE at pH=6 where* B. subtilis *was slightly inhibited (25%), while the inhibition exceeded 44% for* E. coli *under the same conditions. These results highlight the potential use of* A. niger *under nonsterile conditions to be extrapolated at industrial scale for the treatment of tannery effluents.

## Figures and Tables

**Table 1:** Characterization of tannery effluents compared to Tunisian standards for discharges in public canalizations. Values given represent the mean of three replicates.

Parameters (Unit)	Unhairing	Final	NT 106.002
	(UE)	(FE)	
pH	12.13 ± 0.34	11.64 ± 0.53	6.5 – 9
EC (mS/cm)	17.41 ± 0.41	18.2 ± 0.51	—
Salt (g/L)	14.8 ± 0.35	15.47 ± 0.43	—
COD(g/L)	25 ± 2.46	7.2 ± 1.09	1
BOD_5_ (g/L)	2.5 ± 0.68	1.25 ± 0.38	0.4
BOD_5_/COD	0.1 ± 0.02	0.17 ± 0.02	—
TS (g/L)	35.1 ± 1.95	20 ± 1.34	—
TV (g/L)	18.9 ± 1.59	4.9 ± 0.7	—
TSS (g/L)	10.9 ± 0.98	3.37 ± 0.83	0.4
VSS (g/L)	6.1 ± 1.40	2.2 ± 0.64	—
TKN (mg/L)	2632 ± 29.1	448 ±15.34	100
NH_4_^+^- N (mg/L)	572.6 ±19.53	259 ±26.67	100
H_2_S (mg/L)	1020 ±21.45	720 ±24.34	—
Soluble protein (g/L)	8.35 ± 0.72	0.059 ± 0.03	—
Fat (%)	6.62 ± 1.16	1.55 ± 0.34	—
K (mg/L)	8 ± 0.49	194 ±2.33	50
Cr (mg/L)	<< 0.1	7.02 ± 0.76	0.1
Cd (mg/L)	0.018 ± 0.01	0.048 ± 0.01	0.1
Fe (mg/L)	1.046 ± 0.45	1.618 ± 0.23	0.05

Number of samples for each parameter “n” = 3

**Table 2:** Mineral composition of tannery effluents before (T_0_) and after (T_f_) fungal treatment assays compared to Tunisian standards for discharges in public canalizations. Values given represent the mean of three replicates.

	Unhairing effluent (UE)			Final effluent (FE)			NT 106.002
	T_0_	T_f_ (pHi)	T_f_ (pH=6)	T_0_	T_f_ (pHi)	T_f_ (pH=6)	
Cr (mg/L)	—	—	—	1.053 ± 0.27	0.527 ± 0.03	0.375 ± 0.05	*0.1*
BOD_5_ (g/L)	0.375 ± 0.06	0.0954 ± 0.02	0.0375 ± 0.01	0.1875 ± 0.05	0.0834 ± 0.01	0.075 ± 0.01	*0.4*
COD (g/L)	3.64 ± 0.26	1.16 ± 0.17	0.373 ± 0.04	0.69 ± 0.07	0.26 ± 0.07	0.17 ± 0.05	*1*

Number of samples for each parameter “n” = 3

## Data Availability

The “GraphPad” data used to support the findings of this study are included within the article.
